# The Transactions of the American Association

**Published:** 1877-05

**Authors:** 


### Books Reviewed.

*The Transactions of the American Medical Association at its Twenty-seventh Annual Meeting, held in Philadelphia June* 6–9, 1876. Philadelphia: Printed for the Association, 1876.

The present volume of Transactions contains the usual number of addresses, papers, reports etc., and of about the usual order of merit. The first paper which attracts our attention is the addresses of the President, Dr. J. Marion Sims, of New York. This address has received so much criticism at the hands of the medical press that anything which we can now say concerning it has been anticipated. Many of the points which the President takes in his address are well made, but in several others we cannot agree with him. Notably is this the case in regard to the code of ethics and his proposal of a method to place syphilis under control.

The time of the Judicial Council seems to have been taken up with the consideration of the troubles among the profession in Michigan over the Ann Arbor Regular-Homœopathic, or Homœopathic-Regular, School. It seems to us that the following resolution offered by Dr. Toner and adopted by the

Association places it on record in regard to this matter: "*Resolved*, That members of the medical profession who in any way aid or abet the graduation of medical students in irregular or exclusive systems of medicine are deemed thereby to violate the spirit of the ethics of the American Medical Association."

The greatest amount of work seems to have been done in the sections on Medicine, on Public Hygiene and State Medicine, and on Surgery and Anatomy. In the section on Obstetrics and Diseases of Women and Children no paper was presented for consideration, but the admirable address of the chairman afforded ample material for discussion.

The approaching meeting of the International Congress doubtless kept many papers from being read before the Association which would have otherwise been presented. As a whole the volume is every way worthy to be placed beside its predecessors.

